# Magnetic Resonance-Guided High-Intensity Focused Ultrasound Ablation of Uterine Fibroids—Efficiency Assessment with the Use of Dynamic Contrast-Enhanced Magnetic Resonance Imaging and the Potential Role of the Administration of Uterotonic Drugs

**DOI:** 10.3390/diagnostics11040715

**Published:** 2021-04-16

**Authors:** Tomasz Łoziński, Michał Ciebiera, Elżbieta Łuczyńska, Justyna Filipowska, Artur Czekierdowski

**Affiliations:** 1 Department of Obstetrics and Gynecology, Pro-Familia Hospital, 35-302 Rzeszow, Poland; 2 Second Department of Obstetrics and Gynecology, Center of Postgraduate Medical Education, 01-809 Warsaw, Poland; 3 Department of Radiology and Nuclear Medicine, Institute of Medical Sciences, Faculty of Medicine, University of Rzeszow, 35-310 Rzeszow, Poland; ela.luczynska@op.pl (E.Ł.); filipowskajustyna@me.com (J.F.); 4 Department of Radiology, Pro-Familia Hospital, 35-302 Rzeszow, Poland; 5 Department of Gynecological Oncology and Gynecology, Medical University of Lublin, 20-081 Lublin, Poland; arturczekierdowski@umlub.pl

**Keywords:** uterine fibroid, leiomyoma, magnetic resonance-guided high-intensity focused ultrasound (MR-HIFU), diffusion-weighted magnetic resonance imaging (DWI), dynamic contrast-enhanced magnetic resonance imaging (DCE-MRI)

## Abstract

Objective: The assessment of the usefulness of dynamic contrast-enhanced magnetic resonance imaging (DCE-MRI) when qualifying patients with uterine fibroids (UFs) for magnetic resonance-guided high-intensity ultrasound (MR-HIFU). Material and methods: This retrospective, single center study included 283 women who underwent DCE-MRI and were treated with MR-HIFU. The patients were divided according to non-perfused volume (NPV) as well as by the type of curve for patients with a washout curve in the DCE-MRI study and patients without a washout curve. The studied women were assessed in three groups according to the type of uterotonics administered. Group A (57 patients) received one dose of misoprostol/diclofenac transvaginally and group B (71 patients) received oxytocin intravenously prior to the MR-HIFU procedure. The remaining 155 women (group C) were treated with the traditional non-drug enhanced MR-HIFU procedure. Results: The average NPV value was higher in no washout group, and depended on the uterotonics used. Conclusions: We demonstrated a correlation between dynamic contrast enhancement curve types and the therapeutic efficacy of MR-HIFU. Our results suggest that DCE-MRI has the potential to assess treatment outcomes among patients with UFs, and patients with UFs that present with a washout curve may benefit from the use of uterotonic drugs. More studies are required to draw final conclusions.

## 1. Introduction

Uterine fibroids (UFs) are commonly found in women during their reproductive age. Although affecting a high percentage of the female population, the vast majority of these tumors are asymptomatic and do not require an intervention [1,2]. In women with symptomatic UFs and presenting one or more typical symptoms, such as pelvic pressure and pain, heavy, irregular, and prolonged uterine bleeding, or iron deficiency anemia, surgical treatment is usually indicated [3,4]. UF treatment has been changing for years with new drugs or methods appearing and others, less effective or safe, being replaced. The most commonly chosen surgical treatment involves myomectomy or hysterectomy. However, in women who wish to preserve fertility and/or their uterus, surgery might not be the preferred solution [5,6,7]. Other existing treatment options should be directed towards an improvement in symptomatology and the quality of life [8,9]. As regards the conservative interventional treatment options currently available for women with symptomatic UFs, uterine artery embolization (UAE) [10] and focused energy delivery methods are promising but long-term data are still scarce [7,8].

In the last decade, minimally invasive treatment of UFs including magnetic resonance-guided focused ultrasound (MRgFUS) and, later, its more advanced option magnetic resonance-guided high-intensity ultrasound (MR-HIFU) have been introduced [11,12]. This is a non-invasive thermal ablation technique which uses magnetic resonance imaging (MRI) guidance and a focused ultrasound beam applied externally to destroy benign and malignant tumors located in the human body [13]. In the last decade, the successful application of HIFU was described in patients with various malignancies, such as prostate and liver cancers, bone tumors or in selected benign masses, such as UFs [14]. This treatment modality proved to be safe and effective. However, several important limitations of such a type of treatment were also found [15].

**Figure 1:**
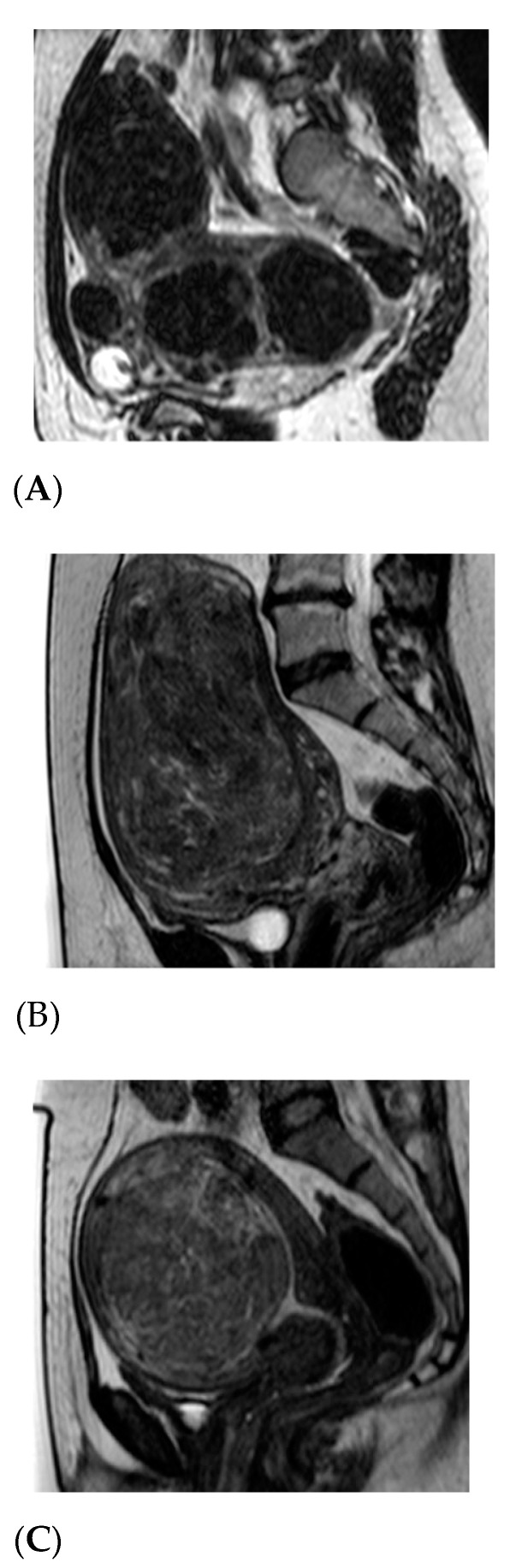
Magnetic resonance imaging (MRI) types of uterine fibroid (UF). (**A**). Type I presents as a “dark” UF as seen on MRI T2-weighted imaging. (**B**). Type II has a mixed MRI bright and dark structure. (**C**). Type III presents in MRI as a “bright” type of UF, usually not suitable for MRI-HIFU (high-intensity ultrasound) treatment.

MR-HIFU therapy uses a special transducer to bundle ultrasound energy into a small volume at the target locations inside the lesion. During treatment, the ultrasound energy beam passes through the intact skin and soft tissue, causing localized high temperatures (between 60–86 °C) only in the tumor. The patient’s skin and intermediate tissue in front of the targeted lesion are left unharmed. Within a few seconds the heat produces a well-defined region of coagulative necrosis [16]. Imaging with 3D MRI provides the anatomical reference data for treatment planning, while real-time temperature sensitive images that are acquired during ablation provide constant real-time information about treatment progress and monitor critical anatomical structures [17,18]. In the case of UFs, the surrounding endometrium may even look as if it was unharmed by the use of this method [19]. The final therapeutic success of MR-HIFU relies on a very careful patient selection in whom such a therapy might be most successful [20]. One of the imaging techniques which enables a clinically useful selection of women with UFs that could be treated with this method is based on T2-weighed MRI. Pretreatment UF signal intensity (SI) and non-perfused volume (NPV) immediately after focused ultrasound independently predicts lesion size reduction at a 12 month-follow up. Funaki et al. proposed diagnostic criteria to distinguish three different types of UF. They are lesions with SI lower than that of skeletal muscles (type I), or lower than that of the endometrium but higher than that of skeletal muscles (type II). In type III SI is higher than that of the endometrium [21]. The examples of the types are provided in Figure 1.

As of today, the best results of MR-HIFU treatment have been demonstrated in women with type I and type II in the MRI-based classification [21,22]. However, this classification is still not optimal and several UFs subjected to MR-HIFU treatment did not react as expected, with resulting suboptimal NPV eventually found after the procedure [23]. In order to increase the success rate various uterotonic agents were proposed [24,25,26,27].

The goal of MR-HIFU tissue ablation is to reduce the symptoms and induce UF shrinkage. Therefore, a complete destruction of the tumor should not be expected and is not the purpose of this type of high-energy focused ultrasound treatment [14]. Although no long-term observation data are currently available, MR-HIFU is roughly comparable to UAE, and appears to be a cost effective treatment option. However, according to Ikink et al. (2014), MR-HIFU might be associated with a higher risk of a re-intervention. Moreover, HIFU is currently regarded as an effective and well-tolerable treatment to be performed as an outpatient procedure without anesthesia or sedation, especially with the use of oxytocin [27,28].

It has been shown that the use of oxytocin or misoprostol during myomectomy or hysterectomy reduces blood flow through the UF and the overall blood loss related to those procedures. However, the evidence to support uterotonics as effective options in reducing blood loss is still of low-quality [29]. The data concerning the use of these drugs, especially those other than oxytocin in MR-HIFU, are still incomplete. The use of these drugs during an MR-HIFU procedure was studied by our team, but from a different angle and on a different issue [27]. The use of a modified oxytocin augmented ultrasound focused energy transmission protocol has recently been proposed as a high-intensity-focused ultrasound (HIFU) treatment for UFs [24,28].

A misoprostol/diclofenac tablet comprises an enteric-coated core of diclofenac sodium (50 mg) surrounded by a mantle of misoprostol (200 mcg). Both agents combined provide powerful anti-inflammatory efficacy. Misoprostol, a prostaglandin analogue, specifically a synthetic prostaglandin E1 (PGE1), is well known, as it causes uterine contractions. It has also been shown to be effective for cervical priming in non-pregnant women prior to hysteroscopy or the insertion of intrauterine devices [30].

**Figure 2:**
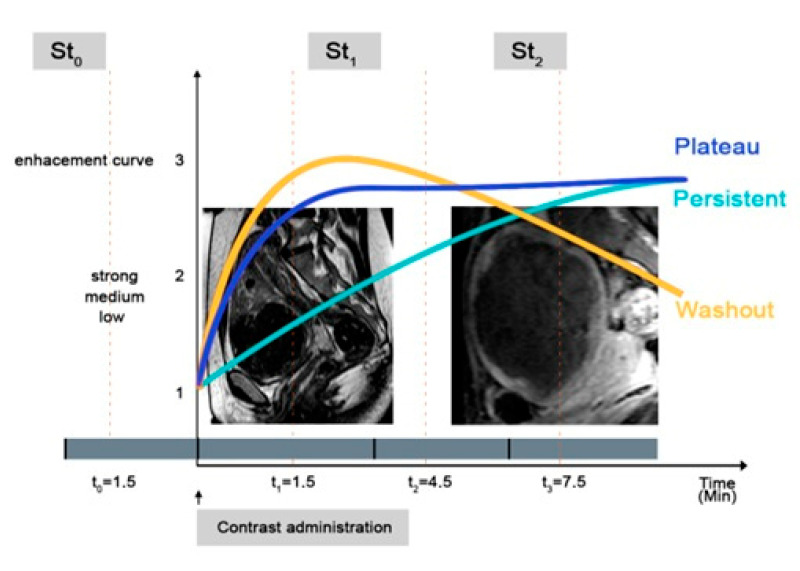
Types of enhancement curve in UFs.

Dynamic contrast-enhanced magnetic resonance imaging (DCE-MRI) enables tumor vascularity to be assessed. By means of the kinetic modeling of signal intensity changes resulting from the passage of a contrast agent through the tumor vasculature, physiologically based parameters can be derived that may reflect tumor perfusion, vascular volume, and angiogenesis [31]. Contrast-enhanced testing is based on differences in the vascularity of the tumor and the surrounding tissues. In 1995, Brown et al. demonstrated the contrast enhancement in T1 images of dependent cancer foci visible shortly after contrast administration [32]. The dynamic examination is based on the observation of the concentration of the contrast substance in the tumor tissue [32]. Three types of gain curves may be distinguished: type 1, with a persistent-steady rise, type 2 (plateau) with a steady increase in the inflow, which flattens after 2 min, and type 3, with washout, where the contrast is quickly washed away [31]. Nowadays, this test is widely used in oncology to diagnose prostate and breast cancer [33]. The examples of gain curves are presented in Figure 2.

The speed of blood flow through the lesion is of importance in the tumor’s response to a focused ultrasound beam. The rapid effect of blood outflowing causes a cooling reaction (takes away the heat), which reduces the effectiveness of the procedure [34].

The aim of the study was to determine if there is a possibility of qualifying patients for MR-HIFU treatment based on DCE-MRI and on the type of enhancement curve in the UF. The second aim was to find out if the parameters would become more accurate and allow exclusion of cases of Funaki types I and II that do not respond well to ultrasound treatment.

## 2. Materials and Methods

### 2.1. Eligibility Criteria

We present a retrospective study basing on the monocentric data. Between April 2015 and February 2020, we identified 688 patients with symptomatic UFs who were potentially eligible for MR-HIFU therapy. This study followed the principles of the Declaration of Helsinki. The study had Local Bioethics Committee Approval and informed written consent was obtained from all the participants. All mandatory laboratory health and safety procedures were complied with in the course of conducting any experimental work reported in this manuscript. The inclusion and exclusion criteria are presented in Table 1.

### 2.2. Interventions

Following the inclusion and exclusion criteria, 283 patients with Funaki type I and type II UFs eventually qualified for this study. All women underwent MR-HIFU treatment in Pro-Familia Hospital, Rzeszów, Poland. A complete HIFU procedure was performed in 232 women. The procedure was discontinued in 51 patients due to pain, the presence of the intestine in the acoustic window and impatience. The UFs were classified into group A (time-SI curve of UF lower than that of the myometrium) and group B (time-SI curve of UF equal to or higher than that of the myometrium). NPV ratios immediately after treatment and UF volume reduction ratios at the 6-month follow-up were retrospectively assessed. The patients were then divided into three groups. Patients in group A (57 women) received one dose of misoprostol/diclofenac and group B (71 women) received oxytocin prior to the HIFU procedure. In the remaining 155 women (group C, controls) the UFs were treated with the traditional non-drug enhanced MR-HIFU procedure. Dynamic contrast enhancement curve types seen on MRI in various types of UFs were recorded. The entire group of treated patients was divided into two groups depending on the obtained NPV (<50% and >50%), and depending on the obtained dynamics of enhancement—with or without a washout curve.

### 2.3. Treatment Procedure

MR-HIFU procedures were performed on an outpatient basis without anesthesia or sedation. Anesthesia or sedation was defined as the use of medications that lower the consciousness level of patients. The use of analgesics does not affect the consciousness level and, therefore, is not regarded as a form of anesthesia or sedation. Treatment sessions were given on a daily basis to cover various parts of the UF until the entire lesion was ablated. The duration of each treatment session lasted no more than 220 min (approx. 220 min in total with the preparation of the patient, documentation, admission to the hospital), and 180 min of sonication.

MR-HIFU procedures were performed with the use of Sonalleve MR-HIFU 3.0 T SN/34009, year of production 2014, Software R3.5L4, MRI Ingenia 3.0T SN /71188 2014 software 5.6.1.2 (Philips, Amsterdam, Netherlands) device with rectum or bladder and rectum manipulation.

The focused ultrasound beam was scanned over the volume to be ablated. The parameters of the apparatus are presented in Table 2.

The urinary bladder was always catheterized and in about 1/3 of cases we used gel rectally to maneuver the uterus. The patients were in a prone position with the lower abdomen submerged in the degassed water of the generator chamber. The entire treatment was performed under no anesthesia or sedation, which meant medications that might lower the consciousness level of the patient were not administered. This allowed constant feedback from the patient throughout the procedure. MR-HIFU was given at a relatively low acoustic intensity of 30–300 W/cm^2^ to allow better patient toleration, for 50 pulses per treatment spot, with an increased transmission time to 400 ms per pulse to enhance heat deposition, a pause time of 100 ms between pulses, with the total treatment time at each spot being 25 s. The treatment was started from the posterior part of the UF in horizontal layers to cover the entire volume of the UF, using a step spacing of 47 mm, a line spacing of 47 mm and a layer spacing of 811 mm. The first sonications were carried out with the use of low power, about 30–40 W, which was gradually increased depending on the tumor response to ultrasound and the temperature achieved.

The full data on the use of the uterotonics is presented in our previous research [27]. Oxytocin group patients were administered 40 IU of intravenous oxytocin diluted in 500 mL of 5% glucose or 0.9% NaCl at the rate of 5 mL/min. Patients included in the misoprostol/diclofenac group were asked to place 2 Pfizer (Bruxelles, Belgium) Arthrotec^®^ pills (a combination of a non-steroidal anti-inflammatory drug (NSAID) and a prostaglandin), with 200 mg misoprostol and 50 mg diclofenac sodium in one pill a total of 400 mg of misoprostol and 100 mg of diclofenac in the two pills) into the vagina posterior vault about 30 min prior to MR-HIFU procedure.

The patients were monitored for blood pressure, pulse rate, oxygen saturation by oximetry, heat sensation tolerance (4-point scale), and pain sensation tolerance (11-point scale). An MRI examination before and after the surgery was performed on T1 and T2 weighted images, T1 FATSAT, T1 after dynamic contrast administration (0.1 mmol/kg Gd-DO3A-butrol, Gadovist; Bayer Schering Pharma (Leverkusen, Germany). The study was evaluated by two highly experienced radiologists. Before the treatment, the contrast enhancement curve from the UF that had been treated at the 1 cm^3^ region of interest (ROI) gate was drawn for every patient. The reinforcement curves were divided into no washout, i.e., constant growth and plateau, as one group and washout as the second group.

### 2.4. Statistical Analysis

All the results were presented in proportions and percentages for categorical variables. They were presented in median and interquartile range (IQR) for continuous variables. The statistical significance of the differences between the study group and the control group were calculated using the Chi^2^ test for categorical variables and using the Mann-Whitney U test for continuous variables. The differences in age, volume change after 6 months, maximal power used, maximal time to optimal temperature, and NPV between three groups were compared with the ANOVA Kruskal–Wallis test. The difference was considered statistically significant with p < 0.05. All analyses were performed using Statistica software (Version 13.0 PL; StatSoft Inc., Tulsa, OK, USA; StatSoft, Krakow, Poland).

## 3. Results

The mean age of the studied group was 35.9 years (range: 20–48 years), median BMI 23.2 kg/m^2^ (range: 15.76–38.3 kg/m^2^), and median NPV-71% (approx. range: 20%–100%). The results are illustrated in Table 3.

The change in UF volume, the maximum temperature obtained in the tumor, the maximum power applied, and the minimum time to optimal temperature are presented in Table 4.

Then, the study group was divided depending on the characteristics of contrast gain and classification according to Funaki et al. [21]. The results are presented in Table 5.

In total, 15% of patients had an unfavorable dynamic enhancement of the washout type, which translated into the effects of the treatment (NPV). In the group of UFs with washout enhancement, the number of treatments with NPV <50% was significantly higher. Moreover, it needs to be emphasized that the procedure was discontinued in 51 patients. This was mainly due to pain, the presence of the intestine in the acoustic window and the uneasiness of the patient. The results are presented in Table 6.

**Figure 3:**
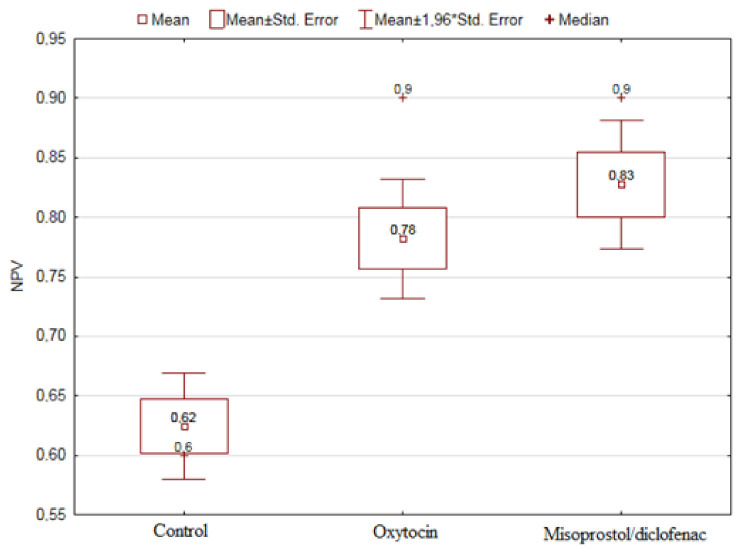
The effect of treatment drugs on NPV in patients with established washout gain curve.

The influence of the applied uterotonic drugs (misoprostol/diclofenac, oxytocin) on the group of patients with an unfavorable washout type of gain in which the treatment effects were worse was examined. The study showed a relationship between NPV and the type of drug administered (*p* = 0.0064). The results are presented in Figure 3.

Importantly, the result shows a strong dependence of NPV on the drugs used in the group of patients with UFs who were found to be washout in the dynamic examination. This confirmed the vasoconstrictor effect of drugs on the non-pregnant uterus and a marked reduction in the cooling effect. These data is in line with our previous research, where we assessed the impact of transvaginal misoprostol/diclofenac and intravenous oxytocin preparation on sonication time and the impact on the peri- and post-procedural effectiveness of misoprostol/diclofenac and oxytocin on MR-HIFU procedure [27].

During our analyses, we also checked the dependence of the type of enhancement curve without washout and with washout on other patient characteristics and treatment parameters such as BMI, age, change in the volume of the UF after 6 months, the maximum temperature reached in the tumor, the maximum power used and the maximum optimal time and temperature in the UF to achieve necrosis. The results are presented below in Table 7.

**Figure 4:**
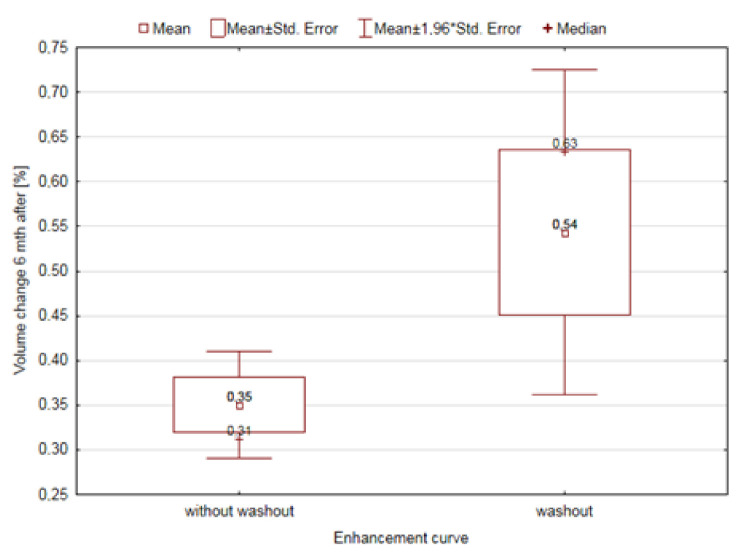
The effect of the distribution of the enhancement curve on the changes in tumor volume after 6 months.

**Figure 5:**
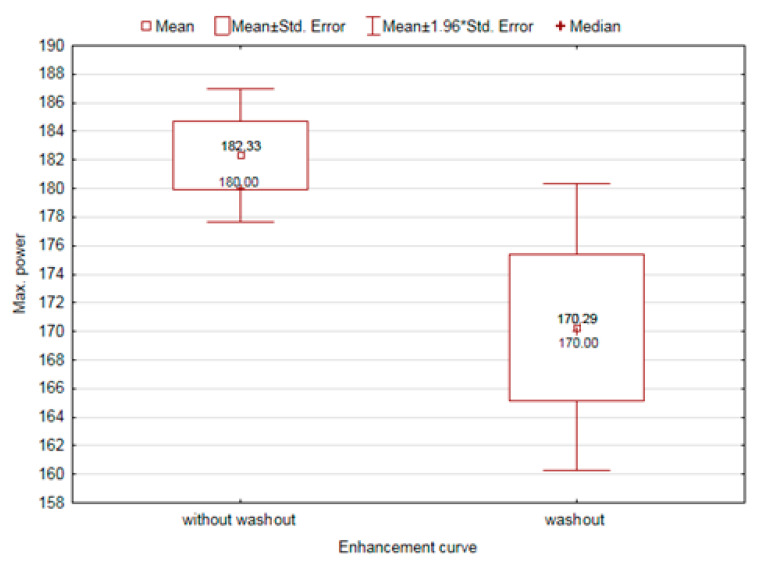
The effect of the distribution of the enhancement curve on the changes in the maximum applied power.

**Figure 6:**
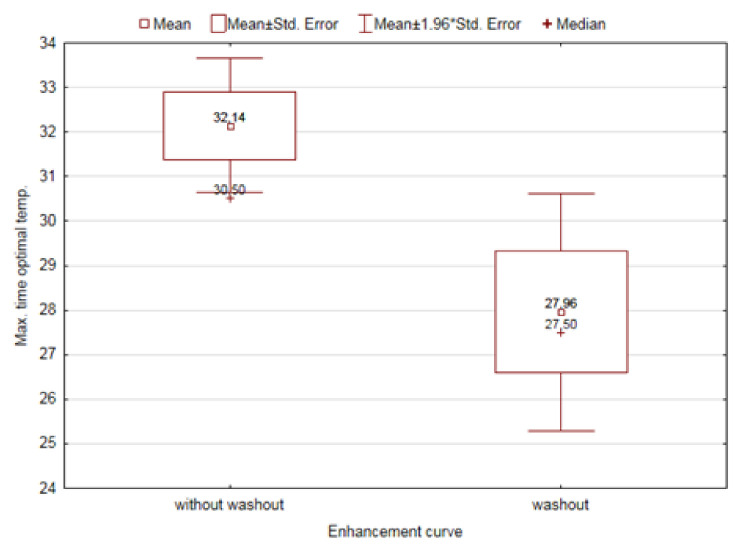
The effect of the distribution of the enhancement curve on the maximum time of optimal temperature.

Parameters that were significantly dependent on the type of the gain curve are additionally illustrated in Figure 4, Figure 5 and Figure 6.

## 4. Discussion

To the best of our knowledge no study on enhancement curves has been conducted so far. The data about the use of oxytocin or misoprostol in MR-HIFU are also recent [24,25,27]. Our study underlines the importance of vascular analysis when qualifying patients with UFs for MR-HIFU procedures. Moreover, a DCE curve type of perfusion-based classification was a strong predictor of treatment outcome. Patient selection is also a significant factor for achieving a high NPV ratio. NPV ratio was strongly correlated with oxytocin or misoprostol use. In our study we used a 3-Tesla (3T) MRI scanning system that facilitated the imaging of high-intensity focused ultrasound tissue effects. Currently, the standard in diagnostics are images obtained by 1.5T scanners. Usually, such an examination is sufficient for the physician to determine whether a disease is underway in a given area of the body. A major advantage of the 3T MRI device is higher resolution images due to the stronger magnet. The higher the value of the magnetic field, the better the image, i.e., the examination can detect more details, and this accounts for the superiority of 3T devices. 3T MRI machines have also been able to shorten scan times without compromising image quality and eliminate patient movements that can distort the image [35]. The advantage of 3T MRI over the most commonly used 1.5T scanners is related to the improved signal-to-noise ratio (SNR) due to greater spin polarization [36]. A higher 3T magnetic field may result in flow artifacts in a dynamic test (greater than in the 1.5T device), but the skillful selection of acquisition parameters allows to minimize these, and we use the higher SI signal strength and better tissue contrast offered by the 3T model. By using the stronger 3 T field, we also use about a 40% stronger signal-to-noise ratio (SNR). The stronger signal obtained in this way can be translated into higher imaging resolution or shorter acquisition time. These features were used in our imaging studies to improve the spatial and/or temporal resolution. We also observed better tissue contrast, including an improved contrast-to-noise ratio (CNR) in most of the studied tumors. Unfortunately, the availability of 3T machines is much more limited than the availability of the standard 1.5T machines and in this case is one of the biggest drawbacks. The lack of access to such systems means that patients might give up this type of examination because they do not want to travel or wait a long time. The cost of performing such a test may also make a difference.

Different opinions are expressed, but some experts believe that MRI outperforms ultrasound and other imaging techniques when HIFU is considered [37]. Contrast-enhanced T1 weighted and T2 weighted imaging is typically employed for treatment planning, monitoring tissue damage and the final result evaluation [38]. In these differentiations MRI may predict the status of contrast enhancing lesions and give results very similar to positron emission tomography with regards to differentiation between tumor recurrence and radiation necrosis [38]. However, the targeted UF non-perfused volume is not directly correlated with the amount of non-viable tumor tissue [39]. One of the possible upgrades of the treatment protocols of MRI imaging for HIFU procedure is the use of uterotonic agents that could restrict the blood flow into the lesion. In our viewpoint and the opinions of other researchers, the proposed treatment modifications could result in a very promising and more favorable long-term clinical outcome that may be superior to those of HIFU alone [28]. Apparently, a randomized control trial at a larger scale is required for further evaluation of this treatment.

An essential element of the correct classification of patients for the MR-HIFU procedure is the earlier determination of the probability of the therapy’s effectiveness [40]. Studies were carried out to compare the features of UFs visible in imaging studies with their structure based on histopathological examinations [41]. On this basis, researchers identified characteristics that are likely to be successfully treated with methods such as MR-HIFU and distinguished them from those that are likely to be less effectively treated. The classification by Funaki et al. is an accepted classification using MRI [21].

The above-mentioned classification does not always predict the final effect of MR-HIFU and symptom regression [42]. The interpretation of T2 weighted images is based on the comparison of the signal values relative to other structures and is subjective. The Funaki classification uses T2 weighted images to determine the type of UFs and, based on this, classifies them into three groups [21]. The signal intensity in the T2 sequences depends on the content of water molecules in the tumor, which may be due to both degenerative processes and the presence of a large number of blood vessels and strong tumor vascularization [20]. The strong vascularization of the UF may cause a poor therapeutic effect of the HIFU procedure due to the significant heat dissipation by the blood flow [20]. For this reason, depending on the radiologists who interpret the examination, the changes are classified into different groups [43]. Therefore, the clinicians who use focused ultrasound therapy procedures constantly look for other, especially quantitative, radiological features of UFs, which would allow for a better prediction of the therapeutic effect of the procedure [7]. Attempts are made to assess changes in multiple parameters (multiparametric MRI) on the basis of the numerical value of the signal intensity in dependent T2 images, dynamic contrast tests and diffusion parameters. However, this is mostly done in other types of lesions, e.g., prostate cancer [44].

Observations suggest that high T2 signal sequence UFs that show late uniform enhancement in imaging studies may be successfully treated with MR-HIFU, as their signal results from high water content rather than strong vascularity [16,45]. Current data suggest that the assessment of the enhancement curves may be an important parameter in the classification of patients for MR-HIFU procedures [46]. So far, based on the evaluation of the described curves, attempts have been made to select the appropriate group of patients for the percutaneous, endovascular UAE [47]. Our results suggest that the evaluation of the dynamic enhancement and the type of the UF enhancement curve should be applied in terms of the potential use of uterotonic drugs during therapy. The proposed new classification method uses the comparison of perfusion-time enhancement curves in the T1-dependent MR images of UF tissue and normal myometrial tissue in the classification of patients for the treatment of UFs. The results of the analysis showed a good correlation between the type of UF determined on the basis of the gain curve and the NPV value. Highly vascularized lesions were more effectively treated by UAE than poorly vascularized ones [48]. The above studies suggest that UFs that are enhanced to a smaller extent than normal myometrial tissue are poorly vascularized, so they can be treated more effectively with MR-HIFU. The so-called cooling effect is interesting in terms of clinical significance. Considering the enormous limitations of UFs in the context of MR-HIFU treatment, reducing this effect gives hope for a greater number of successfully cured patients. Another logical result was the use of higher powers in the group of patients with UFs with washout curves and the time to maintain the optimal temperature in the tumor. The cooling effect of the outflowing blood means that the procedure must be performed with the application of higher parameters, which may be associated with a greater risk of complications, such as skin burns or pain reactions.

The limitation of our work is the analysis of the correlation between the type of vascularization observed in the DCE-MRI as “rapid growth—washout” and the treatment effect of MR-HIFU procedure with this type of UF. The rapid influx and rapid washout of the contrast agent seem to be associated with the strong vascularization of the UF and the rapid blood flow through it. As suggested by researchers, the assessment of vascularization and blood flow through UFs before MR-HIFU procedures is necessary and its results are closely related to the clinical effect of the therapy [14]. Numerous factors influence the therapeutic effect of MR-HIFU. It was noted in histopathological studies that UFs differed in terms of cellularity and the degree of vascularization related to the number of blood vessels within them [49]. It is commonly believed that the presence of a large number of cells and good vascularization result in a poor response to treatment [50]. This may be due to the Pennes Bio-Heat Equation, which allows the prediction of the thermal effect that will occur under the influence of the external energy supplied to the tissue [51]. It seems that tumor vascularization has a greater influence on the effect of HIFU treatment than its cellularity, which was also suggested by Kim et al. [52]. The greater blood flow leads to a faster dissipation of thermal energy from the sonicated area. Therefore, the resultant thermal effect of the treatment may be lower than expected and required [51]. This is somewhat inconsistent with our results concerning 6-month UF volume change. Our results indicate that the UFs shrank more if the gain curve drawn from the UF before surgery was a washout type. However, it should be underlined that the control MRI examination was performed, due to economic reasons, only in patients who achieved > 70% NPV, so this result is only of technical value.

In studies published in recent years, vascular perfusion was mostly assessed by classic MRI with a contrast medium or by semi-quantitative assessment during DCE-MRI [53]. However, these methods are subjective, which may interfere with the correct assessment of vascularity. The results of our research indicate that the use of drugs allows the limitation of the cooling effect by shrinking the tumor. While conducting further research, it is worth considering the use of quantitative tissue perfusion analysis methods currently used in the assessment of malignant neoplasms [54]. Our results allow us to consider conducting research in patients with Funaki group III UFs, who have been completely disqualified so far. Presumably, the administration of uterotonics would facilitate an effective procedure. Further research and development of appropriate criteria seem to be advisable, which, thanks to the perfusion assessment, will make it possible to predict the therapeutic effect of the procedure with high accuracy [7,12].

Based on all the presented data we suggest that the volume changes of ablated UFs, used power and maximum optimal temperature are related in some manner to the type of enhancement curve types found on DCE-MRI and that MRI has the potential to measure hemodynamic changes after MR-HIFU procedure (both with and without uterotonics).

## 5. Conclusions

Our data suggest that the use of DCE-MRI and the assessment of the gain curves increases the accuracy of the qualification examination of patients with symptomatic UFs for MR-HIFU procedures. The indicated method may support a more precise qualification of UF patients, as it may indicate lesions that will present a poor response to treatment, despite promising initial qualification.

Previous studies found that the use of uterotonic drugs might modify the MR-HIFU treatment effect. This study adds that this occurs in patients with UFs that develop a washout curve.

A well-designed prospective study in a larger population of women with different types of UF is required to fully establish the value of DCE-MRI and uterotonic drugs in MR-HIFU.

## Figures and Tables

**Table 1:** Inclusion and exclusion criteria for MR-HIFU procedure.

Inclusion Criteria	Exclusion Criteria
age 20–50 years	pregnancy
UF measuring 1–13 cm in diameter	calcified or predominantly degenerated UFs
blood platelet count ≥ 100,000/mm^3^	obese patients with a thick abdominal wall fat layer
serum creatinine level ≤ 2.0 mg/dL	the presence of a scar in the sonication path
MRI criteria (Funaki type I and II only) optimal acoustic window, i.e., without intestine loops in the sonication path)	contraindications to MRI
maximal number of UFs = 2	pedunculated UFs
	more than 12 cm from the transducer to the front wall of UF
	too short distance from the sacral bone < 20 mm
	The location of the UFs on the back wall of the uterus and/or in direct contact with the rectum

**Table 2:** Acquisition parameters used in Phillips Sonalleve MR-HIFU system.

Parameter	T2 Weighted	Dynamic Contrast Enhanced (DCE) Curves
Acquisition plane	axial, sagittal	turbo field echo imaging
X averages	Non-steroidal anti-inflammatory (NSA)1	NSA1
Slice thickness (mm)	4	2.4
Slice gap (mm)	0.5	−1.2
Acquisition duration	01 min 00 s	4 min 31 s
Phase-encoding direction	anterior-posterior	right-left
Field of view (mm)	230 × 197	320 × 368
Acquisition matrix	256 × 177	320–275
Repetition time/echo time (ms)	1505/80	3.2/1.53
Flip angle (degree)	120	10
Bandwidth (Hz/pixel)	375	723.4

**Table 3:** Characteristics of selected parameters of the studied women and MR-HIFU results (single parameters are missed).

		N	Mean	SD	Min	Max	Q_25_	Median	Q_75_	CV
Age [n]	Control	155	36.19	5.07	19.00	46.00	32.00	37.00	40.00	14.0%
	Misoprostol	56	35.98	4.81	26.00	45.00	32.50	36.50	40.00	13.4%
	Oxytocin	71	35.49	4.63	26.00	48.00	33.00	36.00	38.00	13.0%
BMI [kg/m^2]^	Control	154	23.26	3.47	17.93	34.72	20.58	22.90	25.51	14.9%
	Misoprostol	56	22.87	2.89	16.65	31.31	21.23	22.27	24.21	12.6%
	Oxytocin	71	23.48	4.12	15.76	38.30	20.28	22.65	27.06	17.5%
NPV [%]	Control	116	62.47	24.63	10.00	100.00	50.00	60.00	80.00	39.4%
	Misoprostol	49	82.76	19.20	25.00	100.00	70.00	90.00	100.00	23.2%
	Oxytocin	67	78.21	20.94	20.00	100.00	70.00	90.00	90.00	26.8%
MRI volume [cm^3^]	Control	153	74.62	88.29	2.13	586.50	18.60	42.24	99.10	118.3%
	Misoprostol	54	96.04	113.36	4.64	625.40	20.97	56.30	129.16	118.0%
	Oxytocin	71	92.77	102.50	2.76	550.62	18.65	57.39	139.57	110.5%

BMI—body mass index; NPV—non-perfused volume, CV—coefficient of variation; MRI—magnetic resonance imaging; Q—quartile; SD—standard deviation.

**Table 4:** Selected parameters of the procedure in the study group (single parameters are missed).

	N	Mean	Median	Min	Max	Q_25_	Q_75_	SD	CV
Volume change (6 months)	82	38	36	−26	96	20	60	27	72.03
Max. temperature [°C]	231	78.53	72.60	57.80	178.00	69.00	82.00	17.29	22.02
Max. power [W]	231	179.45	180.00	100.00	270.00	160.00	200.00	33.89	18.89
Min. time to optimal temperature [sec]	146	9.05	8.50	1.00	26.00	5.00	13.00	5.05	55.72

**Table 5:** Types of UF according to the Funaki classification and types of dynamic enhancement in the study group.

	Funaki Type I	Funaki Type II	Total
Washout absent	147 (61.5%)	92 (38.5%)	239 (85%)
Washout present	24 (55%)	20 (45%)	44 (15%)

**Table 6:** NPV and enhancement curve—comparison (total of 232 cases).

NPV	Enhancement Curve		*p*-Value
	No Washout	Washout	
<50%	25 (74%)	9 (26%)	0.0446
>50%	172 (87%)	26 (13%)	

NPV—non-perfused volume.

**Table 7:** Dependence of the type of enhancement curve with and without washout on other patient characteristics and treatment parameters.

Characteristic	Group	Mean	Median	*p*-Value
BMI [kg/m^2^]	Without washout	23.22	22.65	0.7301
	Washout	23.33	23.00	
Age [n]	Without washout	35.88	37.00	0.6563
	Washout	36,47	36.00	
Volume change after 6 months [%]	Without washout	0.35	0.31	0.0085
	Washout	0.54	0.63	
Max. temp [ºC]	Without washout	79.16	72.20	0.9286
	Washout	78.17	73.00	
Max. used power [W]	Without washout	182.33	180.00	0.0407
	Washout	170.28	170.00	
Max. time to optimal temperature [sec]	Without washout	32.14	30.50	0.0186
	Washout	27.96	27.50	

## Data Availability

The data used to support the findings of this study are available from the corresponding author upon request.
